# A queen’s tale: An experimental palaeoproteomic study of a honey bee queen cell specimen from Natural History Museum Denmark

**DOI:** 10.12688/openreseurope.18538.2

**Published:** 2025-07-08

**Authors:** Tuuli Kasso, Meaghan Mackie, Max Ramsøe, Lars Vilhelmsen, Carsten Gundlach, Sina Baier-Stegmaier, Alberto J. Taurozzi, Matthew J. Collins

**Affiliations:** 1 University of Copenhagen, Globe Institute, 1353 Copenhagen, Øster Farimagsgade 5, Denmark; 2 University of Copenhagen, The Novo Nordisk Foundation Center for Protein Research, 2200 Copenhagen, Blegdamsvej 3b, Denmark; 3 Natural History Museum Denmark, SCIENCE, University of Copenhagen, Universitetsparken 15, 2100 Copenhagen, Denmark; 4 Technical University of Denmark, Department of Physics, 2800 Kongens Lyngby, Fysikvej 310, Denmark; 5 University of Cambridge, The McDonald Institute for Archaeological Research, Cambridge CB2 3ER, Downing St., UK

**Keywords:** Apis mellifera, honeybees, queen bee, beeswax, MRJPs, X-ray Computed Tomography, palaeoproteomics, natural history museum collections

## Abstract

**Background:**

Natural history museum specimens of historical honey bees have been successfully used to explore the genomic past of the honey bee, indicating fast and rapid changes between historical and modern specimens, possibly as a response to current challenges. In our study we explore a potential untapped archive from natural history collections - specimens relating to historical beeswax.

**Methods:**

We examine an *Apis mellifera mellifera* queen cell specimen from the 19th century. The intact and closed cell was analysed by X-ray Computed Tomography (CT) to reveal a perfectly preserved queen bee inside her cell. Subsequently, a micro-destructive approach was used to evaluate the possibility of protein extraction for a palaeoproteomic approach.

**Results:**

Using proteomics by liquid-chromatography-tandem mass spectrometry, we were able to recover 120 non-contaminant proteins, mostly bee-related, including major royal jelly proteins (MRJPs) crucial for queen nutrition and development, and silk fibroin proteins. Analysis of the protein modifications indicate their endogenous source and show the presence of *in vivo* glycosylation. This probably helped protein preservation and allows for the study of antimicrobial and metabolic functions of bees in the past.

**Conclusion:**

Our exploratory results show that studies on specimens such as these can provide biomolecular information. Such specimens can inform future research on queen development, diet, and hive conditions, providing valuable context for understanding how honey bee populations have responded to changing environments and pressures over time. This highlights further the scientific potential of natural history collections in addressing contemporary challenges in honey bee conservation and health.

## Plain language summary

This study investigated a 19th-century queen bee cell from a natural history museum to reveal a perfectly preserved queen bee and successfully extracted proteins from the inside of the cell with information about the development and diet of the bee. These findings offer valuable insights into historical bee rearing practices, which can help us better understand and address current challenges faced by honeybees.

## Introduction

Today, natural history museums play a crucial role in advancing scientific knowledge, offering invaluable resources for research into biodiversity, evolution, and climate change. They serve as educational hubs, fostering public understanding and appreciation of the natural world through exhibits and outreach programs. Additionally, these museums are vital for conservation efforts, providing historical baselines to track environmental changes and inform protection strategies for endangered species and habitats.

Therefore, these museum collections can act as reservoirs of biological knowledge, especially since they contain varieties of natural material collected throughout time and space. One such reservoir is the collections of honey bee material that can be relevant to understand changes that have occurred over time to the species, especially those caused by anthropogenic factors. The western honey bee, *Apis mellifera*, is a eusocial insect vital for its irreparable role in ecology, agriculture and economy ( Crane, 1999; Lamei, 2018). Natural history museum collections, with their preserved specimens and historical data, can offer critical insights into the evolution, health, and behaviours of honey bee populations over time. By studying past collections of *Apis mellifera*, researchers can identify genetic diversity, disease patterns, and environmental impacts, aiding in the development of effective conservation strategies in Europe and the rest of the world; this will help to protect and sustain honey bee populations, which are essential for pollination and ecosystem health ( Kasso *et al.*, 2023b; Parejo *et al.*, 2020).

There have been studies successful in extracting DNA from museum specimens of bees ( Lozier & Cameron, 2009; Mikheyev *et al.*, 2015; Parejo *et al.*, 2020; Strange *et al.*, 2009), which is especially important for observing the decrease in genetic diversity that has occurred in honey bees over time ( Espregueira Themudo *et al.*, 2020). Beeswax-containing historical material such as medieval sealing wax, has also been investigated through genomic approaches ( Kasso *et al.*, 2023a; Szulc *et al.*, 2020). As an alternative to ancient DNA, palaeoproteomics explores ancient proteins that could be used to explore questions relating to paleogenomics and microbiology ( Hendy *et al.*, 2018), while also being more likely to survive than DNA over extended time scales. Proteomics have been applied to modern honey bees successfully for insights into development ( Feng *et al.*, 2009; Zheng *et al.*, 2011), pathogens and disease ( Arad *et al.*, 2025; Han *et al.*, 2013) and major royal jelly, including its impact to the development and resistance of bees ( Bíliková *et al.*, 2009; Buttstedt *et al.*, 2014; Drapeau *et al.*, 2006; Mureşan *et al.*, 2022; Zhang *et al.*, 2014b). However, a purely palaeoproteomic approach has not been attempted before.

While each bee has an important role in the colony, it could be valuable to focus research on queen bees specifically. The queen is the only bee without which the entire hive will not survive; she is the mother of all bees in the colony ( Woodward, 2007). Through her pheromones, the colony maintains its order and functions that reflect the requirements of the colony, e.g. the need for foraging ( Crane, 1999; Woodward, 2007). Human led queen rearing (i.e. raising and replacing) has been a prominent practice in beekeeping ever since the 19 ^th^ century, as the quality of the queen significantly affects the whole hive, including its reproductivity and resistance to disease ( Crane, 1999; Woodward, 2007). In Denmark, the bees currently used in beekeeping are a developed hybrid species from 3–4 subspecies of *Apis mellifera*, which has replaced the native bee to the area, *Apis mellifera mellifera*, the European Dark bee (i.e. *den brune bi)*, that has almost disappeared from Denmark ( Nielsdatter *et al.*, 2021).

**Figure 1.**
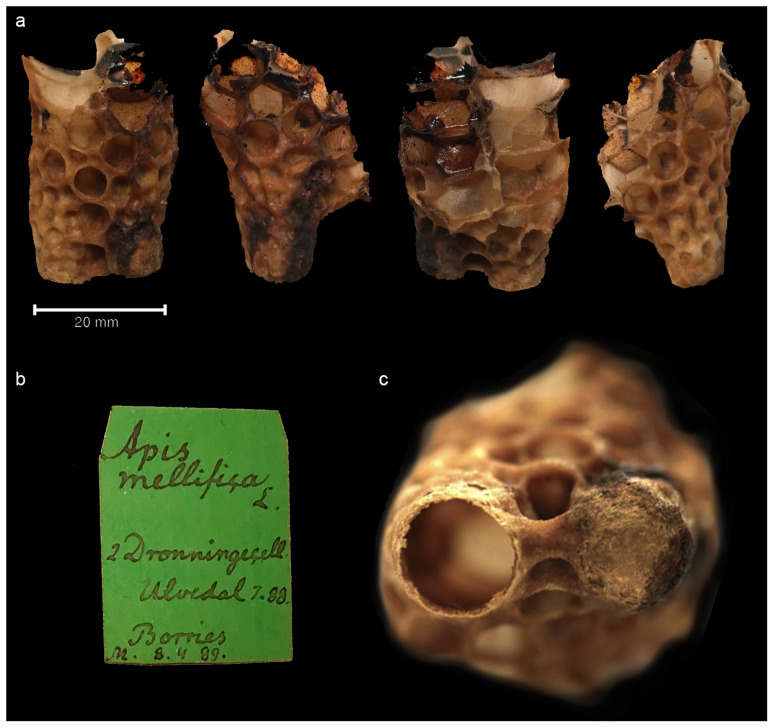
The queen bee cell specimen from all sides. **a**. Piece of honeycomb with queen cell specimen shown from all sides. Figure 1 **b**. Museum label of the specimen. Figure 1 **c**. Close-up of top of the cell (far right): the left one is open and was possibly never occupied, the right one is capped.

In this study, we used both X-ray Computed Tomography (CT) and palaeoproteomics to investigate a queen cell specimen ( Figure 1) from the Natural History Museum Denmark collections, which holds several historical honeycomb specimens. This study was exploratory, to complement the DNA work that has been done on museum bee specimens and to better understand the potential of these techniques applied to historical material. This is especially true of the palaeoproteomics approach, since it has so far not been applied to historical bee specimens. If ancient proteins can be extracted from such specimens, it is feasible that, with more samples, similar work to that applied on modern specimens could be performed on historical specimens to better understand development and disease over time.

An experimental study to extract proteins directly from historical beeswax was also conducted; but due to limited results it is available as Extended Data (ED) (See Data Availability, ED 1).

## Methods

### The queen cell

The specimen ( Figure 1), made accessible by Natural History Museum Denmark, and is estimated to be from the late 19 ^th^ century, based on the hand-written label ( Figure 1b) of the sample, which reads *“Apis mellifica L 2 Dronnningecell Ulvedal 7.88 Borries M 8.4.89”*. “8.4.89” could be interpreted as the date in 1889 when the specimen was collected or recorded by the museum, or it could simply be a catalogue number. Ulvedal could be Ulvedalen in Denmark and Borries the name of the beekeeper - yet no historical archival information was found to support this, so we must leave this to speculation. *“Apis mellifica* L” indicates European Dark bee ( *Apis mellifera mellifera*), which today is rare in Denmark. It was displaced through the introduction of other subspecies and hybrids, such as the Italian *Apis mellifera ligustica*, which was introduced to Denmark ca. 1860 ( Nielsdatter *et al.*, 2021).

The beeswax specimen is formed of two queen cells, with one cell capped ( Figure 1c) and the possibility of having an undisclosed queen inside. Therefore, the specimen was studied with X-ray Computed Tomography (CT) to non-destructively image the interior of the closed cell.

### X-ray computed tomography

The CT-scanning was performed at the 3D Imaging Centre at DTU using a ZEISS Xradia 410 Versa micro CT, which allows a resolution of a few micrometres and typical sample sizes from millimetres to a few centimetres. The maximum power of the instrument is 10 W, and the energy of the employed X-rays can be varied in the range 40–150 keV to optimise contrast that allows for the separation and configuration of different materials, such as the chitinous insect from the lipidic beeswax. The sample was scanned without a filter using 40 kV and 6.6 W, the large field of view objective and a 2x2 detector binning. 3201 projections were acquired with an exposure time of 2 s per projection, leading to a full scanning time of 3 hours. The reconstructed voxel size was 40.6 µm which results in an approximate resolution of 100 µm. During the scanning process, only 2D X-ray images were captured at different angles as the sample was rotated 360 degrees. However, the queen inside the cell was not visible in these images. However, the reconstruction of the 3D volume and the subsequent visualisation using the ThermoFisher Avizo software ( https://www.thermofisher.com/dk/en/home/electron-microscopy/products/software-em-3d-vis/avizo-software.html) allowed the bee to be seen and to segment different components of the image in different colours. Similar analysis and visualisation can be performed with the software tool ITK-SNAP ( www.itksnap.org) ( Yushkevich *et al.*, 2006).

### Palaeoproteomics

**Figure 2.**
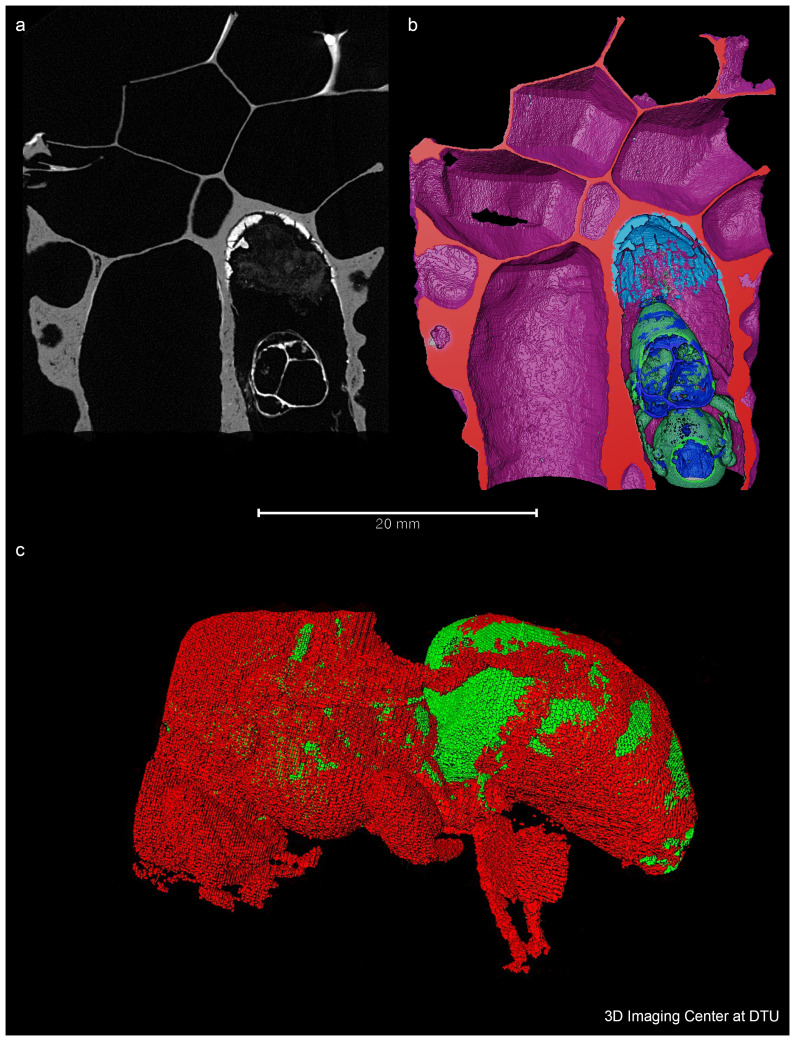
A 2D, 3D volumetric and 3D representation of the bee. **a**. A 2D slice showing one plane of the 3D volume obtained by X-ray CT. The bee is located in the bottom right cell and the residual layer appears bright on the top of this cell, meaning that it has a higher density or is composed of materials with higher atomic number than the material in the darker areas.. This plane is used to virtually cut the volume which enables the view shown in Figure 2 **b**. Figure 2 **b**. A 3D volumetric representation, colour coded according to the identity of the different materials. Note the residual layer (illustrated in light blue) at the bottom of the cell. Figure 2 **c**. 3D representation of the bee isolated from the wax.

From the reconstructed 3D image obtained from X-ray CT, a layer of material can be seen beneath the queen bee (see Figure 2B). It was decided to attempt extracting this material for ancient proteins, as it was considered unethical to sample the queen itself, due to the limited sample size of still encapsulated historic bees. Accessing the queen would have required opening the cell and this would risk damaging or destroying the specimen irreparably, posing limitations for future research to study the morphology of the bee and its genetic material.

### Protein extraction and mass spectrometry

For the micro-destructive extraction of proteins from the queen cell, 100 μl extraction buffer was injected through the wall of the capped cell near the unknown material, using a syringe with a 1mm diameter needle. The extraction buffer was prepared (75:5:4:8:8) with 8M guanidine hydrochloride (Sigma-Aldrich cat. no. G7294), 1M Tris (Invitrogen™ cat. no. 10055704), 0.5M tris(2-carboxyethyl)phosphine) (Sigma-Aldrich cat. no. 646547), 0.5M chloroacetamide (Sigma-Aldrich cat. no. 22790), and ultra-pure H _2_O (AccuGENE™ Molecular Biology Water cat. no. 7732-18-5). It was delicately partially aspirated and dispensed in and out of the cell two times. After this careful resuspension, the extraction buffer was fully aspirated and placed into a 1.5 mL Protein LoBind tube (Eppendorf cat. no. 022431081). An immediate observation was a colour change of the buffer from transparent to translucent dark brown, indicating that the extraction was likely successful in retrieving sample material from inside the cell. The following analysis was performed according to best practice for palaeoproteomic samples to limit modern contamination, including the use of a dedicated laboratory space and nitrile gloves ( Hendy *et al.*, 2018). A blank extraction was also performed to control for laboratory contamination. The sample and blank were first incubated at 80°C for 1h. Protein quantification was made using bicinchoninic acid (BCA) assay Pierce™ BCA Protein Assay Kits cat. no 23225. This confirmed that a sufficient quantity of protein was extracted and available for downstream processing. The sample was then digested first with 1 μl (0.4 μg/μL) of Lys-C (Promega cat. no. V1671) at 37°C for 1 h, followed by digestion with 1 μL trypsin (0.4 μg/μL) trypsin (Promega cat. no. V5111) overnight at 37°C. The purification of peptides was subsequently performed via StageTips ( Rappsilber *et al.*, 2007). The peptides were eluted in 30 μL of 50% acetonitrile (ACN, Thermo Fisher Scientific/Pierce cat. no. 51101) 0.1% trifluoroacetic acid (TFA, Sigma-Aldrich cat. no. T6508).

The samples were analysed using liquid chromatography tandem mass spectrometry (LC-MS/MS) using an EASY nLC 1200 (Proxeon, Odense, Denmark) coupled to a Exploris 480 mass spectrometer (Thermo Scientific, Bremen, Germany), based on methods already published for historical samples ( Mackie *et al.*, 2018). The elutions were vacuum centrifuged at 45°C until approximately 5 µL of sample remained. Samples were then resuspended in 8 µL 5% ACN 0.1% TFA in water. 2 µL of each sample was injected for measurement. The samples were separated on an in-house laser-pulled 15 cm column (75 μm inner diameter, Polymicro Technologies cat. no. TSP075375) and packed with 1.9 μm C18 beads (ReproSil Pur 120 C18-AQ, Dr. Maischcat. no. r119.aq) ) over a 77 min gradient with increasing ACN in 0.1% formic acid (FA, Merck/Supelco cat. no. 5330020050). In short, the MS parameters were as follows: MS1- scan range of 350–1400 m/z, 120k resolution, maximum injection time (IT) of 25 ms, and an AGC target of 300% in Top 10 mode. MS2- 60k resolution, maximum IT of 118 ms, minimum intensity 2e5, AGC target 200%, normalised collision energy of 30%, a dynamic exclusion of 20 s, and an isolation window of 1.2 m/z. To hinder cross-contamination, a wash-blank method using 0.1% TFA, and 5% ACN was run in between each sample.

### Protein identification and modification data analysis

The raw MS/MS data was analysed in two steps. Firstly, a primary screening was made using MaxQuant (v.1.6.3.4, RRID:SCR_014485) ( Cox & Mann, 2008), with a tryptic search of the Swiss-Prot database (downloaded 10/02/22, RRID:SCR_021164) to determine the possible sources of protein in the sample. Search parameters were the defaults for an Orbitrap mass spectrometer. Modifications were as follows: fixed carbamidomethylation of cysteine; variable oxidation of methionine, deamidation of asparagine and glutamine, and pyroglutamic acid of glutamine and glutamic acid. Error tolerances were 10 ppm for the precursor and 0.02 Da for the fragmentions, and the false discovery rate (FDR) was set to 1%. Minimum score cut-off was 60. Identifications from this search led to further searches of the Uniprot (RRID:SCR_002380) honey bee proteome (unspecific digestion search), and a search each for the known Uniprot proteins from *Aspergillus* and *Penicillium* species (trypsin specific), followed by more specific species proteomes with an unspecific digestion search.

All hits from these search paths were then combined into a final database and searched with the same modifications as above but with semi-specific trypsin specificity (max peptide length of 8–25 amino acids), based on the results of the previous searches. The discovered proteins and peptides were then authenticated and identified: proteins with only one peptide detected in the entire dataset were discarded, as well as contaminants clearly deriving from the laboratory process, such as those present in the extraction blank, and from handling, such as keratins. Peptide species specificity was determined by using pBLAST ( Altschul *et al.*, 1990) (RRID:SCR_004870). Deamidation was assessed using publicly available code ( https://github.com/dblyon/deamidation) ( Mackie *et al.*, 2018).

In order to examine other post-translational modifications (PTMs), another search was made with this database using PEAKS (version 7.5, RRID:SCR_022841), to utilise the PEAKS PTM module ( Han *et al.*, 2011) to find unspecified modifications. The results of this search identified N-glycosylation sites, specifically N-Acetylhexosamine, which were confirmed with another MaxQuant search with this as a variable modification.

## Results and discussion

### X-ray CT

The CT investigation successfully resulted in locating the queen and 3D images were generated of the bee that looks well developed ( Figure 2). The formation stages of the queen bee from a *larvae* to a queen are well known ( Woodward, 2007), and the adult queen usually emerges from the cell around the 16th day. As both the development and hatching of the bee needs a temperature of 35°C ( Woodward, 2007) maintained by the worker bees, removing this cell from the beehive and keeping it at a lower temperature likely terminated the development and caused the death of the queen. However, pathogens may also have caused disease and impacted the queen.

From the reconstructed 3D image obtained, a layer of material can be seen beneath the queen bee (see Figure 2B). In order to better characterise this layer, a palaeoproteomic workflow was performed on the material, using a minimally destructive method that avoided opening the cell completely.

### Palaeoproteomics

**Figure 3.**
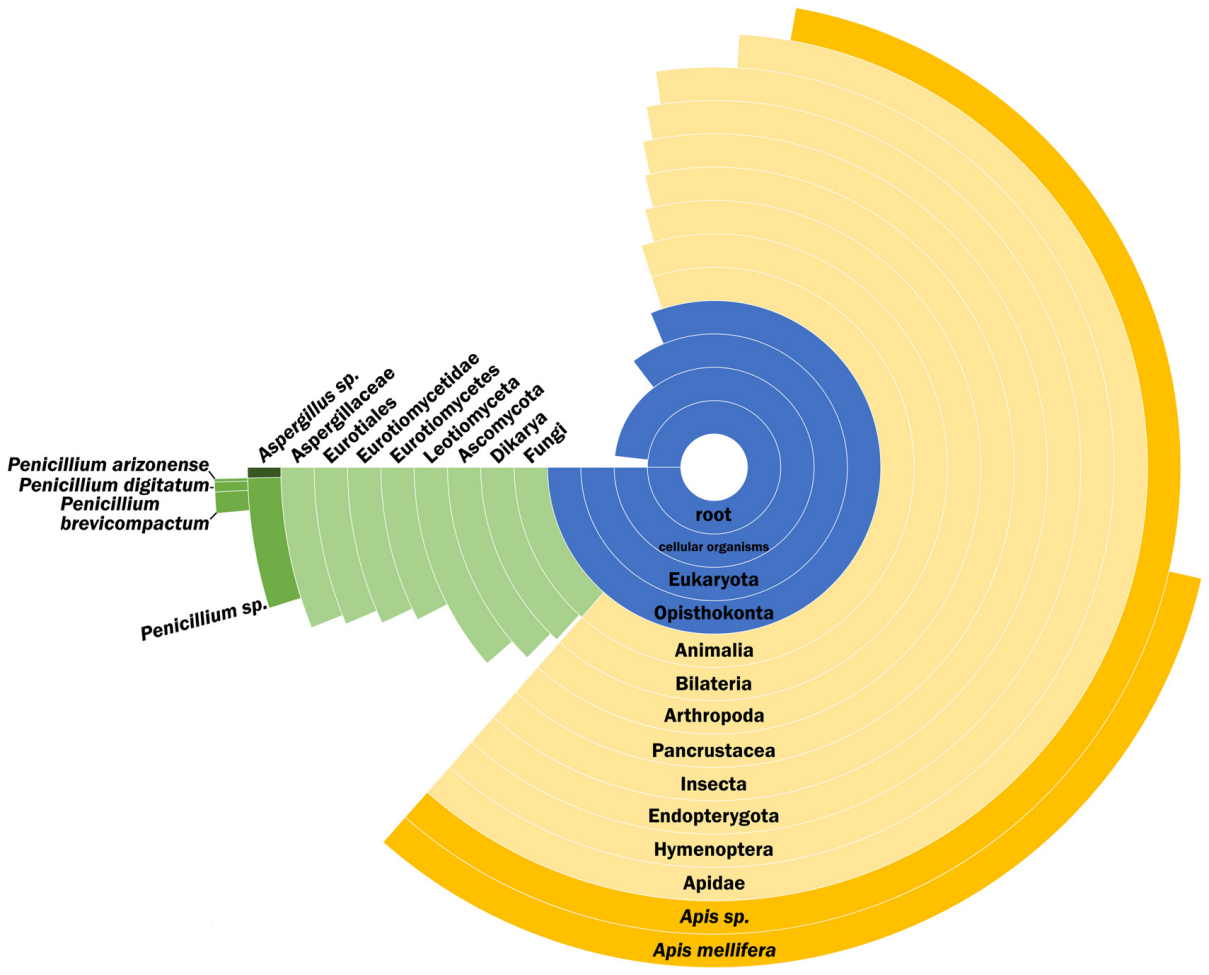
Peptide Species Specificity distribution of the sample. Peptide Species Specificity distribution of the sample after removal of lab reagent contamination and proteins reported with only one peptide. Percentage of the circle of each group indicates the percentage of peptides specific to that group.

We successfully identified 120 proteins (ED 2) from the material retrieved from the encapsulated cell sample, with the most relevant groups being bee-related proteins ( Figure 3). The most abundant proteins are mostly represented by major royal jelly proteins (MRJPs) and silk fibroin proteins. In addition, proteins from *Penicillium* and *Aspergillus* species (ED 2) were also recovered. It is clear that the unknown material seen in the X-ray CT scan is mostly composed of MRJPs and silk fibroins, left behind from the development of the queen. During this development, queens are fed MRJPs and the queen larvae makes a silk cocoon for itself, covering the cells ( Hepburn *et al.*, 2013; Micas *et al.*, 2016). There are four different types of bee silk proteins ( Micas *et al.*, 2016), and we identified all of them in our data. Due to the presence of other proteins, such as enzymes, it is likely that some of this material also represents faecal or other deposital material from the bee, such as the meconium (i.e. the gut contents that are voided as the bee emerges from the pupa).

Many of the royal jelly proteins were recovered. There is no inherent difference between the eggs which develop into workers and queens. The only difference is the nutrition provided, with the prospective queens fed royal jelly ( Buttstedt *et al.*, 2014; Woodward, 2007), which is more nutritious than the standard worker fare. Royal jelly (or ‘bee milk’) is high in both protein and carbohydrates and triggers the queen’s development; its quality is critical for this event, despite the actual biological mechanism behind it not being completely understood ( Buttstedt *et al.*, 2014; Crane, 1999; Woodward, 2007). MRJPs form up to 15% of the royal jelly that is fed to the queen during its development and its life ( Buttstedt *et al.*, 2014; Woodward, 2007). We were able to identify five of the nine MRJPs known (MRJP 1, 2, 3, 5 and 7), and these are also the most abundant ones in royal jelly ( Buttstedt *et al.*, 2014), explaining the better coverage of them in our sample.

In addition to its nutritional value, royal jelly is considered to be both antifungal and antimicrobial ( Shen *et al.*, 2012). Some *Apis* specific enzymes found were Defensin-1 and glucose oxidase. Defensin-1 is found in royal jelly ( Bucekova *et al.*, 2017), and in low concentrations it acts as a mechanism against gram-positive bacteria, e.g. *Paenibacillus larvae*, the cause of American Foulbrood disease ( Ryba *et al.*, 2009; Shen *et al.*, 2012). Related to antimicrobial properties, we were also able to detect N-Acetylhexosamine glycosylation at several sites in MRJPs 1 and 2 (ED 3) three of which have been predicted to occur from sequence analysis (see UniProt Knowledgebase, https://www.uniprot.org, for these proteins). In addition, there was spectral evidence for a previously unrecorded hexose modification site on MRJP3. Some other sites were detected with varying confidence (ED 3), and more research needs to be done to confirm their presence. Their presence is important here because this modification has shown to give these proteins antimicrobial effects ( Bíliková *et al.*, 2009; Mureşan *et al.*, 2022). In MRJPs, it is also related to metabolic activities important for the high metabolic fuel demands of an egg-laying queen ( Zhang *et al.*, 2014a). This modification also may increase the stability of the proteins ( Lis & Sharon, 1993), which may explain why it has been found in modern and ancient bones, as well as ancient eggshell ( Cleland, 2018; Demarchi *et al.*, 2016; Schroeter, 2024).

While the modern bee proteome contains thousands of proteins, we have recovered comparatively few. This is probably due to both the nature of the sample and its age. Over time, the degradation of proteins from environmental conditions (and possibly enzymes present from the bee itself) likely limited the recovery to those that were more abundant or more stable. The sample is also likely not representative of the bee proteome anyway, as the sample was only taken from around the bee. Finally, it is possible that wax or some other substance was extracted along with the proteins that caused interference with the trypsin digestion and/or the mass spectrometry, and limited the peptides recovered. However, there were still several proteins extracted that can give some information about the development of the bee and the environment of the queen cell. It is also of note that this project was much more successful than a similar approach applied to beeswax itself, which only provided minimal proteomic evidence of royal jelly proteins and only from wax from queen cell specimens (See ED 1).

Other non-bee related proteins were also recovered, specifically to *Penicillium* and *Aspergillus* species. The microbial environment of the hive is a delicate balance that protects the bees against pathogens, including fungi. The fungi *Penicillium* and *Aspergillus* are present in the natural environment, therefore also present in the hive as well. *Penicillium* and *Aspergillus* species also thrive indoors and are associated with dust and/or biodeterioration of museum pieces, therefore, they could originate from the sample being from a (natural) museum environment. In fact, some of the specific species detected have been detected in indoor Danish dust ( Andersen *et al.*, 2021; Frisvad & Gravesen, 1994), although it is obviously not specific to Denmark. However, dust contamination is somewhat unlikely as the cell itself was closed, and the area outside cleaned prior to perforating the wall of the specimen for extraction. Also, no contaminant keratin proteins were recovered from the sample, only the extraction blank, indicating limited dust presence in the sample. It is possible that the fungi proteins are endogenous to the sample, as honey bees also collect fungal spores for nutrition ( Parish *et al.*, 2020). Therefore, another advantage of using closed cells in future biomolecular studies of historical bees is the isolated environment that may reflect a pristine proxy for the original developmental environment of the queen bee.

In addition, the deamidation rate tells us about the preservation and the relative age of the proteins from the samples. This modification occurs to the amino acids asparagine (N) and glutamine (Q) over time. Older, and therefore more likely to be endogenous proteins, should have higher deamidation. The contamination present (mostly human keratins in the laboratory blank) has much lower rates of deamidation than those relating to the actual sample (mean of 3.6% for N and 4.3% for Q in the contamination; compared to 14.5% and 23.9%, respectively, for the queen sample), supporting that the proteins from the actual sample are endogenous to the queen cell (ED 4). Since this specimen is from the 19th century, this level of damage is consistent with the historical age, compared to archaeological samples ( Ramsøe *et al.*, 2021; Schroeter & Cleland, 2016). However, it really only shows that they are similarly damaged, as deamidation cannot really be used as an age indicator ( Ramsøe *et al.*, 2021; Schroeter & Cleland, 2016).It is also of note that the fungi and bee samples seem equally damaged (deamidation mean of 13.6% for N and 29.9% for Q for the fungi), despite the bee samples appearing to have more unspecific hydrolytic cleavage (61.4% semi-tryptically cleaved peptides versus 32.0%, ED 4). This cleavage also occurs over time with the breakdown of proteins due to hydrolysis, and is associated with older samples ( Cappellini *et al.*, 2018; Schroeter & Cleland, 2016). The similar deamidation levels could indicate that the fungal and bee proteins are relatively the same age, therefore the fungi proteins could be from an earlier source than dust contamination. The higher rate of unspecific hydrolytic cleavage in the bee proteins might then be from enzymes from the digestive tract of the bee itself.

## Conclusions

In this study, we used both X-ray Computed Tomography (CT) and palaeoproteomics to investigate a queen cell specimen from the Natural History Museum Denmark in order to better understand the specimen and evaluate the possibility of biomolecular (protein) extraction to gain biological insight to past bee rearing. 

With the help of X-ray CT, the bee and the residue of its developmental environment were identified in a closed cell. This allowed us to carefully identify and place the optimum sampling location for maximum information with as little intervention to the bee as possible through micro-destructive sampling. This allows maximal information retrieved with limited damage to the queen.

We were successful in extracting proteins from the residual material inside the queen cell, identifying several bee related proteins, including major royal jelly proteins (MRJPs). (MRJPs). The MRJPs and defensive enzymes are interesting from the perspective of the development and nutrition of the queen, in addition to the protective microbiome of the hive against pathogens. The detection of glycosylation sites informs on antimicrobial properties of MRJPs in the past. Discovering and imaging the fully preserved queen in conjunction with the proteomics data will allow comparisons with modern samples. These studies, in conjunction with further work on other historic or ancient samples, could inform on changes in queen bee development over time.

This experimental study of a queen cell from the Natural History Museum Denmark brings forward the profound potential these collections hold. This small study not only shows that proteinaceous material can be recovered from such a specimen, but that it can be a source of information to better understand bees in the historic past. It can illuminate aspects of the queen bee’s diet (MRJPs, potentially fungal spores) and antimicrobial protection of the cell environment, but it also demonstrates how such preserved specimens in general can serve as time capsules for future scientific research. In contrast to historical beeswax specimens, which we attempted unsatisfactorily (ED 1), closed cells are an excellent source of proteins and potentially other biomolecules. Their preservation and isolation from the environment renders these samples the most promising for future studies on historical bees and could be a future direction or application for this type of targeted sampling. Future research integrating both DNA and proteomic analyses of more samples could provide a more comprehensive understanding of historical honey bee populations, including potential subspecies identification and their adaptive responses to past environmental conditions.

## Ethics and consent

Ethical approval and consent were not required.

## Data Availability

EMBL-EBI :PRIDE database: Assessing the hidden potential of beeswax specimens in Natural History Museum collections. The underlying data has been deposited in the ProteomeXchange Consortium via the PRIDE partner repository, accession number PXD034106: https://www.ebi.ac.uk/pride/archive/projects/PXD034106 ( Mackie, 2024). Data are available under the terms of the Creative Commons Public Domain license (CC0). EMBL-EBI :PRIDE database: Assessing the hidden potential of beeswax specimens in Natural History Museum collections. https://www.ebi.ac.uk/pride/archive/projects/PXD034106 ( Mackie, 2024). This project contains the following extended data: Extended_Data_2_Queen_Cell_protein_tables.xlsx Extended_Data_1-3-4.pdf Data are available under the terms of the Creative Commons Public Domain license (CC0).
