# Supplementary material for: A queen’s tale: An experimental palaeoproteomic study of a honey bee queen cell specimen from Natural History Museum Denmark
Source: Open Res Eur. 2025 Jul 8;4:227. Originally published 2024 Oct 18. [Version 2] doi: 10.12688/openreseurope.18538.2 (PMC12344410; doi:10.12688/openreseurope.18538.2)
Supplement: Peer review history

# Reviewer report: Reviewer response for version 2

**DOI:** 10.21956/openreseurope.21939.r56542

**Published:** 2025-08-29

**Authors:** Richard Bernstein

**Affiliations:** 1 Institute for Bee Research Hohen Neuendorf, Hohen Neuendorf, Germany

The suggestions from my earlier report were incorporated into the manuscript or reasons were provided why this is not possible.

 The authors added more background on Proteomics in honeybees and other studies of ancient materials from honeybees in the Introduction. In the Results & Discussion, the manuscript now mentinons the limitations of working with ancient material for proteomics more clearly.  

 The authors explained in their response that a comparison of their results to studies of current bees would not be fruitful, as changes over time in their samples and the bees are probably large, and their results are of limited amount. A larger sample number or optimisation of the retrieval method could change this situation. 

 Work on museum samples is challenging, and the study successfully retrieved proteomic data from museum samples.

Is the study design appropriate and does the work have academic merit?

Yes

Is the work clearly and accurately presented and does it cite the current literature?

Partly

If applicable, is the statistical analysis and its interpretation appropriate?

Not applicable

Are all the source data underlying the results available to ensure full reproducibility?

Yes

Are the conclusions drawn adequately supported by the results?

Yes

Are sufficient details of methods and analysis provided to allow replication by others?

Yes

Reviewer Expertise:

population genetics of honeybees based on DNA data

I confirm that I have read this submission and believe that I have an appropriate level of expertise to confirm that it is of an acceptable scientific standard.

# Reviewer report: Reviewer response for version 2

**DOI:** 10.21956/openreseurope.21939.r56539

**Published:** 2025-08-12

**Authors:** Nelson Enrique Arenas

**Affiliations:** 1 Universidad de Cartagena, Cartagena, Bolívar, Colombia

The authors addressed accurately all comments.

Is the study design appropriate and does the work have academic merit?

Yes

Is the work clearly and accurately presented and does it cite the current literature?

Partly

If applicable, is the statistical analysis and its interpretation appropriate?

Yes

Are all the source data underlying the results available to ensure full reproducibility?

Partly

Are the conclusions drawn adequately supported by the results?

Yes

Are sufficient details of methods and analysis provided to allow replication by others?

No

Reviewer Expertise:

NA

I confirm that I have read this submission and believe that I have an appropriate level of expertise to confirm that it is of an acceptable scientific standard.

# Reviewer report: Reviewer response for version 1

**DOI:** 10.21956/openreseurope.20042.r47994

**Published:** 2024-12-24

**Authors:** Richard Bernstein

**Affiliations:** 1 Institute for Bee Research Hohen Neuendorf, Hohen Neuendorf, Germany

The authors studied a single queen from the 19th century collected in Denmark. The analysis of museum samples is very challenging, but could yield very interesting information. The authors focus on palaeoproteomics which is a rather new method compared to DNA studies. The sequences of the identified proteins are accessible in the extended data, which is beneficial for future research.

 The main success of the study probably lies in the identification of some proteins from the challenging sample material. At least to my knowledge, proteomics on ancient bee samples have not been done yet, although genomic studies of bee samples from the 19th century have been done. Compared to the number of proteins in a honeybee, only a small amount could be identified, namely 120 proteins. Results and Discussion focus on specific proteins, and explain their role in honeybees in general, or why some finds might be due to dust contamination.

 For honeybee biology in general, only the identified sequences are interesting, but a comparison of honeybees in the 19th century to current ones is not presented in this study. The authors merely suggest that such an comparison may be possible in the future. Such comparisons are possible with DNA analyses of museum samples [1]. With DNA data from the queen, we could e.g. check whether the sample was from *Apis mellifera mellifera *or another subspecies of *Apis mellifera*.

 I criticize that the results are about methods in paleoproteomics, and not highly relevant to the biology of honeybees. E.g. the history of honeybees in Denmark is mentioned in the Introduction, but not relevant for the Results and Discussion. I suggest that the effectivity of the method should be discussed, and a comparison with other methods to investigate historic honeybee populations should be included. In particular, the current study does not cite any previous paleoproteomic work in honeybees. The authors do provide literature on paleoproteomics, but this is not sufficient. There are certainly studies which speculate about the history of honeybee proteins [2]. If it is the first study of this kind, then the authors should mention it. Another important question would be, how well the proteomic analysis of museum samples performed compared to proteomic analysis of recent samples.

Is the study design appropriate and does the work have academic merit?

Yes

Is the work clearly and accurately presented and does it cite the current literature?

Partly

If applicable, is the statistical analysis and its interpretation appropriate?

Not applicable

Are all the source data underlying the results available to ensure full reproducibility?

Yes

Are the conclusions drawn adequately supported by the results?

Yes

Are sufficient details of methods and analysis provided to allow replication by others?

Yes

Reviewer Expertise:

population genetics of honeybees based on DNA data

I confirm that I have read this submission and believe that I have an appropriate level of expertise to confirm that it is of an acceptable scientific standard, however I have significant reservations, as outlined above.

## References

[1] : Digging into the Genomic Past of Swiss Honey Bees by Whole-Genome Sequencing Museum Specimens. *Genome Biol Evol* .2020;12(12) : 10.1093/gbe/evaa188 2535-2551 10.1093/gbe/evaa188 32877519 PMC7720081

[2] : Evolution of the Yellow/Major Royal Jelly Protein family and the emergence of social behavior in honey bees. *Genome Res* .2006;16(11) : 10.1101/gr.5012006 1385-94 10.1101/gr.5012006 17065613 PMC1626640

# Response

**Published:** 2025-04-24

**Authors:** Tuuli Kasso

**Affiliations:** University of Copenhagen Globe Institute, Copenhagen, Capital Region of Denmark, Denmark

We kindly thank the reviewer for the detailed comments and helpful suggestions that have significantly improved our paper. Following is our responses in a point-by-point manner (in bold):   The authors studied a single queen from the 19th century collected in Denmark. The analysis of museum samples is very challenging, but could yield very interesting information. The authors focus on palaeoproteomics which is a rather new method compared to DNA studies. The sequences of the identified proteins are accessible in the extended data, which is beneficial for future research. The main success of the study probably lies in the identification of some proteins from the challenging sample material. At least to my knowledge, proteomics on ancient bee samples have not been done yet, although genomic studies of bee samples from the 19th century have been done. Compared to the number of proteins in a honey bee, only a small amount could be identified, namely 120 proteins. Results and Discussion focus on specific proteins, and explain their role in honey bees in general, or why some finds might be due to dust contamination. Response:. We have rephrased and revised the paper on Results and Discussion, especially in adding a part that talks about the limited protein retrieval. We have not gone too in depth on the proteins present, as we do not want to overinterpret at this point in time, but more indicate the retrieval of protein from this kind of specimen is possible.    For honey bee biology in general, only the identified sequences are interesting, but a comparison of honey bees in the 19th century to current ones is not presented in this study. The authors merely suggest that such an comparison may be possible in the future. Such comparisons are possible with DNA analyses of museum samples [1]. With DNA data from the queen, we could e.g. check whether the sample was from Apis mellifera mellifera or another subspecies of Apis mellifera. Response: We acknowledge that our study does not directly compare historical and modern honey bee proteomes. As mentioned in response to another reviewer, there is no real comparable data to use at the moment. Any abundance patterns when compared to even the same kind of sample from modern times would be fraught with uncertainty since each protein would degrade differently based on its composition/structure and the conditions in which it was kept. Understanding changes in abundances over time would certainly be interesting, but it would need many more samples and is out of the scope of this exploratory study.

 Our primary aim was to explore whether proteins could be retrieved from a 19th-century queen cell and what insights this might offer about historical bee biology. While DNA studies have provided direct comparisons of genetic variation over time, our work demonstrates that palaeoproteomics could complement these studies by revealing protein expression patterns. In general, more samples will need to be taken to make any useful comparisons. We started with one sample, to limit the destruction of irreplaceable samples in case it was not successful. Method development could also be possible to optimise the retrieval of proteins. We have revised  the discussion to more explicitly state that future research could integrate DNA and proteomic data to gain a deeper understanding of historical honey bee populations, including subspecies identification.   I criticize that the results are about methods in paleoproteomics, and not highly relevant to the biology of honeybees. E.g. the history of honeybees in Denmark is mentioned in the Introduction, but not relevant for the Results and Discussion. I suggest that the effectivity of the method should be discussed, and a comparison with other methods to investigate historic honeybee populations should be included. In particular, the current study does not cite any previous paleoproteomic work in honeybees. The authors do provide literature on paleoproteomics, but this is not sufficient. There are certainly studies which speculate about the history of honeybee proteins [2]. If it is the first study of this kind, then the authors should mention it. Another important question would be, how well the proteomic analysis of museum samples performed compared to proteomic analysis of recent samples. Response: We appreciate the reviewer’s feedback and agree that a more detailed discussion of method effectiveness and comparative approaches would strengthen the manuscript. We expanded our discussion to address the advantages and limitations of palaeoproteomics, and have revised our literature on proteomics to honey bees, albeit there has not been previous studies on applied palaeoproteomics on honey bees. Additionally, our data is from the inside materials of the queen cell, therefore we acknowledge that our data is not as representative of the bee, more more so of her environment. We have refined this in the paper, to better align with the study’s findings.

# Reviewer report: Reviewer response for version 1

**DOI:** 10.21956/openreseurope.20042.r46924

**Published:** 2024-12-14

**Authors:** Simon Hammann

**Affiliations:** 1 University of Hohenheim, Stuttgart, Germany

In their article “A queen’s tale: Assessing the hidden potential of beeswax specimens in Natural History Museum collections” the authors describe the analysis of a queen bee beeswax cell specimen from the 19 ^th^ century from a museum collection. The authors emphasise how museum collections can be a valuable source of information. They used µCT to visualise the position of the queen bee and extracted a part of additional material found in the cell. Using LC-MS/MS – based proteomics they identified the material to be consistent with royal jelly, but they also found fungal proteins. Deamidation patterns suggested the royal jelly proteins and the fungal proteins to be contemporary, suggesting it was not a modern contamination. They paper also mentions the extraction of proteins from beeswax itself, but these data are not presented in the main paper.

 I like the general premise of the paper to tap into these rich resources in Natural History Museums. However, I feel the paper is lacking a clear hypothesis or research question. The paper is also written a bit vague in the introduction section (“If the queen was present, proteomics would…”). Furthermore, the first and second paragraph feel unconnected, as there is a very sudden jump from museums to bees. Please try to make this a bit more cohesive. Also, I suggest to emphasise the specific research questions for proteomics part a bit more (third paragraph): What information could be gathered through proteomics potentially? How does this refer to paleogenomics? What questions exactly could be answered here.

 The introduction ends with a reference to the extraction of proteins from beeswax itself and that this is not presented in the main manuscript. Looking at the supplementary material it seems that this part is in length and data richness almost comparable to the main manuscript. I do not really understand why the authors mention this, but then hide it away in the Extended Data (which is generally not reviewed). In my opinion this is not extended data for the main manuscript but a whole different paper/dataset (with its own introduction and Experimental section!). If the authors want to publish this, it should be included into the main manuscript and subject to proper review. If not, I suggest the authors delete this completely from this submission.

 Specific comments:

 Method section, line 11: Should this be 40 keV?

 “During the scanning…”: This sentence is quite complicated. Please consider rephrasing.

 Results and Discussion: When talking about the deamidation damage, it would be nice to have the numbers in the main text and not only a reference to the ED

 Since I am not an expert on proteomics I can’t fully evaluate the quality of the proteomics results. But with the reported sequence coverage of the MRJP species the identification of the material as royal jelly looks very reasonable to me.

 Generally, the results and discussion section feels short compared to Introduction and Methods. Figure 2, currently cited in the introduction, should be moved to the results and discussion. Figure 2 also shows the intact bee, which also doesn’t work with the speculation about the presence of the queen in the penultimate paragraph of the introduction.

 In summary, I believe the authors have a nice little case study highlighting the potential to access these specimens on a larger scale. I believe the manuscript could be improved by emphasising more what exactly could be learned from doing so. I also believe, the publication of the beeswax part of the paper as unreviewed extended data is inappropriate and should be reconsidered.

Is the study design appropriate and does the work have academic merit?

Yes

Is the work clearly and accurately presented and does it cite the current literature?

Partly

If applicable, is the statistical analysis and its interpretation appropriate?

Not applicable

Are all the source data underlying the results available to ensure full reproducibility?

Yes

Are the conclusions drawn adequately supported by the results?

Partly

Are sufficient details of methods and analysis provided to allow replication by others?

Yes

Reviewer Expertise:

Food chemistry, Lipid analysis, Organic Residue Analysis

I confirm that I have read this submission and believe that I have an appropriate level of expertise to state that I do not consider it to be of an acceptable scientific standard, for reasons outlined above.

# Response

**Published:** 2025-04-24

**Authors:** Tuuli Kasso

**Affiliations:** University of Copenhagen Globe Institute, Copenhagen, Capital Region of Denmark, Denmark

We kindly thank the reviewer for the detailed comments and helpful suggestions that have significantly improved our paper. Following is our responses in a point-by-point manner, in bold:   In their article “A queen’s tale: Assessing the hidden potential of beeswax specimens in Natural History Museum collections” the authors describe the analysis of a queen bee beeswax cell specimen from the 19th century from a museum collection. The authors emphasise how museum collections can be a valuable source of information. They used µCT to visualise the position of the queen bee and extracted a part of additional material found in the cell. Using LC-MS/MS – based proteomics they identified the material to be consistent with royal jelly, but they also found fungal proteins. Deamidation patterns suggested the royal jelly proteins and the fungal proteins to be contemporary, suggesting it was not a modern contamination. They paper also mentions the extraction of proteins from beeswax itself, but these data are not presented in the main paper.  

 I like the general premise of the paper to tap into these rich resources in Natural History Museums. However, I feel the paper is lacking a clear hypothesis or research question. The paper is also written a bit vague in the introduction section (“If the queen was present, proteomics would…”). Furthermore, the first and second paragraph feel unconnected, as there is a very sudden jump from museums to bees. Please try to make this a bit more cohesive. Also, I suggest to emphasise the specific research questions for proteomics part a bit more (third paragraph): What information could be gathered through proteomics potentially? How does this refer to paleogenomics? What questions exactly could be answered here.

**Response:** We thank the reviewer for constructive feedback on the introduction to the manuscript/ project. In order to connect paragraph 1 and 2, we have added a new section to highlight the importance of bee collections in natural history museums.

 We have also strived to clarify more of the research questions. In the third paragraph we have added examples of how proteomics have been applied to modern honey bee specimens, and what questions those solve. We have also specified in the 5th paragraph the exploratory nature of this work, to see if palaeoproteomics is a viable route in the future to better understand bee development and disease overtime.   

 The introduction ends with a reference to the extraction of proteins from beeswax itself and that this is not presented in the main manuscript. Looking at the supplementary material it seems that this part is in length and data richness almost comparable to the main manuscript. I do not really understand why the authors mention this, but then hide it away in the Extended Data (which is generally not reviewed). In my opinion this is not extended data for the main manuscript but a whole different paper/dataset (with its own introduction and Experimental section!). If the authors want to publish this, it should be included into the main manuscript and subject to proper review. If not, I suggest the authors delete this completely from this submission.

**Response:** Thank you for your feedback. We appreciate your perspective on the beeswax analysis report and the role of supplementary materials. Our intent in mentioning the beeswax analysis was to acknowledge that we attempted protein extraction from this material, rather than to present it as a central part of the study. Unfortunately, it did not work, and it is important that this information is available to other researchers so they do not waste precious, irreplaceable, historic material on a similar method.  We recognize the importance of ensuring that supplementary materials are scientifically and properly reviewed, therefore we also cite it in our paper and have provided it as an open source (since Open Research Europe does not allow a supplementary), so that it can be reviewed along with the manuscript and other supplementary material. We strongly believe that supplementary data can contribute valuable context to a study, and we believe it also brings transparency.

 We do not want to delete ED1 for these reasons, and in order to remind the reader of its existence and to quickly summarise the results and their relevance of to this project, we have added the following in the main manuscript:  “It is also of note that this project was much more successful than a similar approach applied to beeswax itself, which only provided minimal proteomic evidence of royal jelly proteins and only from wax from queen cell specimens (See Data Availability, ED 1).”  

 Specific comments: Method section, line 11: Should this be 40 keV? “During the scanning…”: This sentence is quite complicated. Please consider rephrasing.

**Response:** We have corrected the typo and rephrased the text.

 Results and Discussion: When talking about the deamidation damage, it would be nice to have the numbers in the main text and not only a reference to the ED

**Response:** We have added the % of deamidation and semi-tryptically cleaved peptides to the main text to better illustrate the damage without looking at the ED.   

 Since I am not an expert on proteomics I can’t fully evaluate the quality of the proteomics results. But with the reported sequence coverage of the MRJP species the identification of the material as royal jelly looks very reasonable to me. Generally, the results and discussion section feels short compared to Introduction and Methods. Figure 2, currently cited in the introduction, should be moved to the results and discussion. Figure 2 also shows the intact bee, which also doesn’t work with the speculation about the presence of the queen in the penultimate paragraph of the introduction.  

**Response:** We appreciate your assessment of our proteomics results, particularly regarding the identification of royal jelly proteins. We acknowledge that the Results and Discussion section was relatively concise compared to the Introduction and Methods, and we have revised it to ensure a more balanced presentation of our findings. Regarding Figure 2, we agree that it would be more appropriately placed within the Results and Discussion section, where it directly supports our analysis, and it has been moved. Additionally, the introduction was refined to ensure consistency in our description of the queen’s presence, aligning it more clearly with the evidence presented in the results.  

 In summary, I believe the authors have a nice little case study highlighting the potential to access these specimens on a larger scale. I believe the manuscript could be improved by emphasising more what exactly could be learned from doing so. I also believe, the publication of the beeswax part of the paper as unreviewed extended data is inappropriate and should be reconsidered.  

**Response:** Thank you for your thoughtful and encouraging feedback. We appreciate your recognition of our study’s potential to inspire broader research on historical specimens. We have worked to strengthen our discussion to better highlight the insights that could be gained from scaling up this approach. Regarding the report on beeswax analysis, we have decided not to include it in the main paper, as we feel it would dilute the focus of our core findings. Our intent was simply to acknowledge that we attempted protein extraction from beeswax, as our results were not useful. We do not wish to pursue the report as a full scientific paper, but still provide all the necessary data, in case it is of use to future study, and would expect it to also be reviewed for content

# Reviewer report: Reviewer response for version 1

**DOI:** 10.21956/openreseurope.20042.r45859

**Published:** 2024-12-14

**Authors:** Luca Fontanesi

**Affiliations:** 1 University of Bologna, Bologna, Italy

This study investigated a queen cell specimen approx. 150 years old collected from a natural museum in Denmark. The study is simple as just one specimen was analysed using X-ray CT and paleoproteomics.

 A few corrections are needed:

 1) the title is misleading, it should be changed indication what was done and the specific objective/results of the study.

 2) Introduction: "...while also being more likely to preserve than DNA.". This is not completely true.

 3) Methods: the following sentence is not clear and should be re-phased: " *Apis mellifica* L indicates European dark bees, which today as a honeybee subspecies is rare in Denmark due to its displacement by other subspecies and hybrids, such as the Italian *Apis mellifera ligustica* which was introduced to Denmark ca. 1860 ( Nielsdatter *et al.*, 2021)."

 4) Methods: It is not clear the meaning of  "... It was decided to experiment with extracting this material for ancient proteins to learn about this specimen, as it was considered unethical to sample the queen itself." Ethical issues in this context are not relevant.

 5) It not completely clear how the queen cell was accessed.

 6) Methods: please refine the way in which numbers and subsequent metrics are written - numbers should be separated from metric acronyms - and then be consistent in writing L derived measures using the same acronym: uL and not ul

 7) Results: it is not clear if from the results or the information acquired on the specimen it is possible to obtain an indication on how old is the analysed queen cell material.

 8) Results: no relevant results were obtained - it seems that the proteomics analyses did not identify anything unexpected. It would be good to list some of the most relevant proteins detected and add a table for that.

 9) Conclusions. this sentence is not correct or is too bold: "These studies could inform on changes in queen be development over time, such as possible changes and developments in major royal jelly proteins." it is actually one study and I doubt that there could be any information useful to evaluate changes of development and the possibility to estimate differences of composition of the royal jelly compared to what we have now.

 10) It is not clear how "This small study not only illuminates aspects of the queen bee’s diet and hive conditions …" This study could not detect any relevant results for this.

 11) I wonder how many capped queen cells could be studied from museum specimens to have such a high expectation

 12) DNA analyses of this specimens should be mentioned as one of the most important net step

Is the study design appropriate and does the work have academic merit?

Yes

Is the work clearly and accurately presented and does it cite the current literature?

Partly

If applicable, is the statistical analysis and its interpretation appropriate?

Not applicable

Are all the source data underlying the results available to ensure full reproducibility?

Yes

Are the conclusions drawn adequately supported by the results?

Partly

Are sufficient details of methods and analysis provided to allow replication by others?

Partly

Reviewer Expertise:

Apiculture, animal genetics, eDNA, animal food research and authentication

I confirm that I have read this submission and believe that I have an appropriate level of expertise to confirm that it is of an acceptable scientific standard, however I have significant reservations, as outlined above.

# Response

**Published:** 2025-04-24

**Authors:** Tuuli Kasso

**Affiliations:** University of Copenhagen Globe Institute, Copenhagen, Capital Region of Denmark, Denmark

We kindly thank the reviewer for the detailed comments and helpful suggestions that have significantly improved our paper. Following is our responses in a point-by-point manner, in bold:   This study investigated a queen cell specimen approx. 150 years old collected from a natural museum in Denmark. The study is simple as just one specimen was analysed using X-ray CT and paleoproteomics. A few corrections are needed:

 1) the title is misleading, it should be changed indication what was done and the specific objective/results of the study. Response: We have reformulated the title.

 2) Introduction: "...while also being more likely to preserve than DNA.". This is not completely true. Response: While there could be cases where DNA preserved better than proteins, it is well established in the ancient biomolecule community that proteins are more stable over longer time scales than ancient DNA due to their chemical and physical properties (see Hendy, J. et al. (2018) ‘A guide to ancient protein studies’, Nature Ecology & Evolution, 2(5), pp. 791–799., and Demarchi, B. et al. (2016) ‘Protein sequences bound to mineral surfaces persist into deep time’, eLife, 5, p. e17092. And also studies where proteins have been recovered where DNA has not, for example : Welker, F. et al. (2015) ‘Ancient proteins resolve the evolutionary history of Darwin’s South American ungulates’, Nature, 522(7554), pp. 81–84.; Cappellini, E. et al. (2019) ‘Early Pleistocene enamel proteome from Dmanisi resolves Stephanorhinus phylogeny’, Nature, 574(7776), pp. 103–107. However, we have reformulated the sentence to make this more clear.

 3) Methods: the following sentence is not clear and should be re-phased: "Apis mellifica L indicates European dark bees, which today as a honey bee subspecies is rare in Denmark due to its displacement by other subspecies and hybrids, such as the Italian Apis mellifera ligustica which was introduced to Denmark ca. 1860 (Nielsdatter et al., 2021)." Response: This sentence has been amended.   

 4) Methods: It is not clear the meaning of  "... It was decided to experiment with extracting this material for ancient proteins to learn about this specimen, as it was considered unethical to sample the queen itself." Ethical issues in this context are not relevant. Response: We would argue that the sampling of historical material always has an element of ethical issues. Since these samples are irreplaceable, the amount of damage done must be worth the information gained. In this situation, the fully formed queen still in a closed cell was determined to be too rare to cause that much damage, but the material in the cell was considered okay to sample from. In order to make this more clear, we have added “, due to the limited sample size of still encapsulated historic bees.” at the end of the sentence.  

 5) It not completely clear how the queen cell was accessed. Response: We have clarified accessing the specimen in the collections and also added a note to the acknowledgements.  

 6) Methods: please refine the way in which numbers and subsequent metrics are written - numbers should be separated from metric acronyms - and then be consistent in writing L derived measures using the same acronym: uL and not ul Response: The inconsistencies in the metric reporting have been fixed.  

 7) Results: it is not clear if from the results or the information acquired on the specimen it is possible to obtain an indication on how old is the analysed queen cell material. Response: Unfortunately, protein damage does not give a precise indication on the age of a sample, since this is influenced by many factors. We know the specimen is from the 19th century, and the damage is consistent with this. Finding the exact age of the sample was not a research question, however, we did attempt radiocarbon dating of the beeswax of the specimen, with no better resolution for the date (data not shown).  

 8) Results: no relevant results were obtained - it seems that the proteomics analyses did not identify anything unexpected. It would be good to list some of the most relevant proteins detected and add a table for that. Response: For future research, there is a full list of the recovered proteins in the extended data (ED 2), accessible on the PRIDE upload https:// www.ebi.ac.uk/pride/archive/projects/PXD034106. We mention major royal jelly proteins (MRJPs) and silk fibroin proteins, and have since reworded this to show that most of the abundant proteins fall into either of these groups. The material seen in the X-ray CT scan was unknown and we were able to identify it as material left behind from the development of the queen, and we have added a sentence to make this more clear. The Penicillium and Aspergillus proteins and the glycosylation of the MRJPs were unexpected findings that have also contributed to our interpretation of the material, as mentioned in the discussion.    

 9) Conclusions. this sentence is not correct or is too bold: "These studies could inform on changes in queen be development over time, such as possible changes and developments in major royal jelly proteins." it is actually one study and I doubt that there could be any information useful to evaluate changes of development and the possibility to estimate differences of composition of the royal jelly compared to what we have now. Response: This sentence has been amended.  

 10) It is not clear how "This small study not only illuminates aspects of the queen bee’s diet and hive conditions …" This study could not detect any relevant results for this. Response: This sentence has been amended to make it more clear.  

 11) I wonder how many capped queen cells could be studied from museum specimens to have such a high expectation Response: Indeed, and we understand that in our case, the sample may be a one-off study. However, we hope that our investigation may give new ideas for different lines of research on Natural History Museum collections.  

 12) DNA analyses of this specimens should be mentioned as one of the most important net step Response: Unfortunately, ancient DNA (aDNA) analysis on leftover extraction buffer was attempted, but with no success. We have not included it in this study in order to streamline the story. We have also attempted aDNA analysis from beeswax (Kasso, T.M., Enevold, R., Johns, S. et al. ArcHives—combined palynological, genomic and lipid analysis of medieval wax seals. Herit Sci 11, 11 (2023). https://doi.org/10.1186/s40494-022-00848-6), and it similarly did not provide sufficient information.

# Reviewer report: Reviewer response for version 1

**DOI:** 10.21956/openreseurope.20042.r45481

**Published:** 2024-11-08

**Authors:** Nelson Enrique Arenas

**Affiliations:** 1 Universidad de Cartagena, Cartagena, Bolívar, Colombia

The study presented in the natural history of *A. mellifera* specimens from the Natural History Museum collection is quite relevant to understanding the biology of pollinators through ancient times.

 Abstract: This section must be strengthened since the findings are interesting, but its abstract is the weakest.  Methods and results are not described in detail.

 Introduction: Authors should connect paragraphs to present the background of the study sequentially. For example, one paragraph describes honeybees, and the next Natural History Museum collections are without any context. Perhaps check how to order the whole story more connected and add the study aims at the end of this section. Authors should describe better if there is any other study in the field of palaeoproteomics with a similar approach.

 The fifth paragraph must be moved to the “Results and Discussion” section including Figures 1 and 2.

 Methods: In the section “Palaeoproteomics” separate the data analysis or bioinformatic methods from the experimental settings. I also recommend to define a little longer subtitle for each section.

 Results and discussion: The CT´s results appeared in the introduction section and should be moved here. I suggest including a list of the proteins identified as supplementary material despite that no differences were found. Authors must include a major information regarding the fold change of MRJPs and perhaps comparing with other honeybees’ species or consider to review if abundances have any explanation through ancient times.

 Since the *A. mellifera*´s proteome has thousands of proteins, authors must explain why this low number of proteins, if any technical problems at obtaining clean protein fractions which is difficult due to exoskeleton or complex polysaccharides, wax or lipid content, or some specific inhibitors.

 The last part of the technical issues in sample processing was not clear.

 Conclusions: The authors must summarize this section.

 The manuscript must be proofread by a native speaker.

Is the study design appropriate and does the work have academic merit?

Yes

Is the work clearly and accurately presented and does it cite the current literature?

Partly

If applicable, is the statistical analysis and its interpretation appropriate?

Yes

Are all the source data underlying the results available to ensure full reproducibility?

Partly

Are the conclusions drawn adequately supported by the results?

Yes

Are sufficient details of methods and analysis provided to allow replication by others?

No

Reviewer Expertise:

Biochemistry, molecular biology, bioinformatics.

I confirm that I have read this submission and believe that I have an appropriate level of expertise to confirm that it is of an acceptable scientific standard, however I have significant reservations, as outlined above.

# Response

**Published:** 2025-04-24

**Authors:** Tuuli Kasso

**Affiliations:** University of Copenhagen Globe Institute, Copenhagen, Capital Region of Denmark, Denmark

We kindly thank the reviewer for the detailed comments and helpful suggestions that have significantly improved our paper.

 Following is our responses in a point-by-point manner, in bold: The study presented in the natural history of A. mellifera specimens from the Natural History Museum collection is quite relevant to understanding the biology of pollinators through ancient times.

 Abstract: This section must be strengthened since the findings are interesting, but its abstract is the weakest.  Methods and results are not described in detail.

 Response: We have added to the abstract to provide more detail in response to this and the other reviewers.  

 Introduction: Authors should connect paragraphs to present the background of the study sequentially. For example, one paragraph describes honey bees, and the next Natural History Museum collections are without any context. Perhaps check how to order the whole story more connected and add the study aims at the end of this section. Authors should describe better if there is any other study in the field of palaeoproteomics with a similar approach. Response: The introduction has been restructured according to all the comments from reviewers.  

 The fifth paragraph must be moved to the “Results and Discussion” section including Figures 1 and 2.

 Response: Most of the fifth paragraph has been removed or moved to the more appropriate sections, as suggested. We have kept Figure 1 in the materials section, as it is not a result of the methodology, but Figure 2 is still in the results section.    

 Methods: In the section “Palaeoproteomics” separate the data analysis or bioinformatic methods from the experimental settings. I also recommend to define a little longer subtitle for each section.

 Response: We have added the sections “Protein Extraction and Mass Spectrometry” and “Protein identification and Modification Data Analysis”.  

 Results and discussion: The CT´s results appeared in the introduction section and should be moved here. 

 Response: We have removed any results from the introduction.  

 I suggest including a list of the proteins identified as supplementary material despite that no differences were found. Authors must include a major information regarding the fold change of MRJPs and perhaps comparing with other honey bees’ species or consider to review if abundances have any explanation through ancient times.  

 Response: We have already provided a list of the proteins identified in the extended data on the PRIDE upload (ED2), since Open Research Europe does not allow supplementary files to be uploaded. We are not sure what the reviewer is referring to when mentioning differences or fold change, since we are not comparing our recovered proteins to anything else. There is no real comparable data to use. Any abundance patterns when compared to even the same kind of sample from modern times would be fraught with uncertainty since each protein would degrade differently based on its composition/structure and the conditions in which it was kept. Understanding changes in abundances over time would certainly be interesting, but it would need many more samples and is out of the scope of this exploratory study.  

 Since the A. mellifera´s proteome has thousands of proteins, authors must explain why this low number of proteins, if any technical problems at obtaining clean protein fractions which is difficult due to exoskeleton or complex polysaccharides, wax or lipid content, or some specific inhibitors.  

 Response: We have added a paragraph to the discussion about the limits of our protein recovery.

 The last part of the technical issues in sample processing was not clear.

 Response:  We are unsure what the reviewer is referencing here, as we do not discuss sample processing issues in the results and discussion. We discuss the possibility of proteins coming from contaminant sources, but clarify that the Penicillium and Aspergillus peptides are possibly endogenous and the bee proteins we recover are likely also endogenous due their level of protein damage (deamidation).  

 Conclusions: The authors must summarize this section.

 Response: This section has been condensed to better conclude the manuscript.  

 The manuscript must be proofread by a native speaker.

 Response: We have had the manuscript proofread by a native speaker, including some of the authors.
